# Anorexia, pain and peripheral neuropathy are associated with a decrease in quality of life in patients with advanced pancreatic cancer receiving outpatient chemotherapy — a retrospective observational study

**DOI:** 10.1186/s40780-021-00210-1

**Published:** 2021-08-02

**Authors:** Hironori Fujii, Maaya Koda, Shiori Sadaka, Koichi Ohata, Hiroko Kato-Hayashi, Hirotoshi Iihara, Ryo Kobayashi, Takuma Ishihara, Shinya Uemura, Takuji Iwashita, Hideki Hayashi, Tadashi Sugiyama, Masahito Shimizu, Akio Suzuki

**Affiliations:** 1 Department of Pharmacy, Gifu University Hospital, Yanagido 1-1, Gifu, 501-1194 Japan; 2 Laboratory of Pharmacy Practice and Social Science, Gifu Pharmaceutical University, Gifu, 501-1196 Japan; 3 Gifu University Hospital, Innovative and Clinical Research Promotion Center, Gifu University, Gifu, 501-1194 Japan; 4 First Department of Internal Medicine, Gifu University Hospital, Gifu, 501-1194 Japan

**Keywords:** Quality of life, Pancreatic cancer, Adverse events related outpatient cancer chemotherapy, Proportional odds logistic regression model, Retrospective observational study

## Abstract

**Background:**

Cancer chemotherapy usually improves clinical outcomes in patients with advanced pancreatic cancer (APC), but can also cause moderate-to-severe adverse events (AEs). We investigated the relationship between moderate-to-severe AEs and quality of life (QOL) in patients with APC who received outpatient chemotherapy.

**Methods:**

We recruited APC patients who received outpatient chemotherapy in Gifu University Hospital between September 2017 and December 2018. Adverse events related to chemotherapy were assessed by a pharmacist collaborating with a physician using common terminology criteria for AEs (CTCAE) ver 4.0, and QOL of patients was self-assessed by patients using the five-level EuroQol five-dimensional questionnaire (EQ-5D-5L Japanese edition 2). Associations between the EQ-5D-5L utility value and serious AEs were assessed using proportional odds logistic regression.

**Results:**

A total of 59 patients who received 475 chemotherapy cycles were included. The proportional odds logistic regression indicated that grade ≥ 2 anorexia, pain and peripheral neuropathy were significantly correlated with a decreased EQ-5D-5L utility value. Pharmaceutical intervention for these AEs significantly improved the patients’ EQ-5D-5L utility value.

**Conclusions:**

Anorexia, pain and peripheral neuropathy were significantly associated with a decrease in QOL. It is assumed that appropriate pharmaceutical intervention with particular emphasis on these AEs can improve the QOL of pancreatic cancer patients receiving outpatient chemotherapy.

## Background

Pancreatic cancer has an extremely poor prognosis, the worst among many malignancies’ five-year (7.0% for males and 5.9% for females) and ten-year (4.6% for males and 4.8% for females) survival rates [1].

In patients with unresectable recurrent pancreatic cancer, first-line chemotherapeutic regimens such as oxaliplatin + irinotecan + fluorouracil + levofolinate calcium (FOLFIRINOX) or gemcitabine + nab-paclitaxel (GnP) are recommended [2–5]. Gemcitabine monotherapy, gemcitabine + erlotinib, or S-1 are recommended as treatment options for patients with poor performance status [6, 7]. FOLFIRINOX therapy and GnP therapy exhibited clinical superiority over gemcitabine monotherapy with respect to overall survival (OS), progression-free survival (PFS), and tumor response rate (RR) in patients with metastatic pancreatic cancer; however, severe adverse events (AEs) such as neuropathy, nausea/vomiting, diarrhea, fatigue, alopecia, and neutropenia were more frequent with FOLFIRINOX or GnP than with gemcitabine [2–5]. The severity and frequency of AEs associated with chemotherapy for pancreatic cancer is generally seen as strongly related to the quality of life (QOL) of patients.

Cancer chemotherapy has been shifting from an inpatient to outpatient setting because of advancements in supportive care. Patients can spend time at home during cancer treatment, but at the same time, they may experience chemotherapy-induced AEs at home and need to manage them by themselves. Chemotherapy for pancreatic cancer, such as FOLFIRINOX therapy and GnP therapy, is often administered in an outpatient setting; thus, appropriate monitoring and supportive care are required. We have reported that QOL tends to be lower in patients with pancreatic cancer than in those with the other cancers [8]. Identifying which AEs cause deterioration in QOL is useful to maintain the patient’s QOL.

Several reports have described the effect of adverse events causing a decrease in QOL in patients receiving chemotherapy for cancer types other than pancreatic cancer [9, 10]. Tachi et al. showed that chemotherapy-induced AEs such as anorexia significantly reduced the QOL of breast cancer patients [9]. In addition, Mark et al. [10] reported that health-related QOL was decreased in patients with advanced-stage lung cancer who experienced strong negative emotions related to side effects, and recommended proactive management of low-grade AEs to improve health-related QOL. Furthermore, APC is often associated with abdominal pain, which can reduce a patient health-related quality of life [11]. However, it is unclear which AEs reduce QOL in patients undergoing chemotherapy for pancreatic cancer.

In this study, we investigated the association between AEs and deterioration in QOL for patients with APC receiving outpatient chemotherapy, and evaluated the effects of pharmaceutical interventions to improve QOL.

## Methods

### Patients

The study was conducted using a retrospective observational design. All patients receiving outpatient cancer chemotherapy for APC were recruited at Gifu University Hospital, Japan, between September 2017 and December 2018 for this study. No inclusion/exclusion criteria were established because all patients with pancreatic cancer who had been treated in an outpatient chemotherapy unit during a defined period of time were considered eligible. We extracted the utility values of QOL, type and severity of AEs, chemotherapy regimens, and other patient demographics using electronic medical records.

### Assessment of AEs

AEs such as anorexia, nausea, diarrhea, oral mucositis, dysgeusia, peripheral sensory neuropathy, pain, arthralgia, myalgia, malaise, and alopecia were assessed by pharmacists and nurses, and corroborated with a physician. The AEs were graded based on the Common Terminology Criteria for Adverse Events (CTCAE) version 4.0 [12]. We noted the highest grade that patients experienced between the previous chemotherapy cycle and the day when the QOL was evaluated. AEs ≥ Grade 2 were considered to be serious.

### QOL assessment

The Japanese edition of the EQ-5D-5L developed by Shiroiwa et al. [13] was used in face-to-face interviews to estimate the utility values of QOL [13] and was routinely implemented at the start of each chemotherapy cycle.

The EQ-5D-5L includes five subsections, including mobility, self-care, usual activities, pain/discomfort, and anxiety/depression. Each of those includes five levels of severity: level 1, no problem; level 2, slight problem; level 3, moderate problem; level 4, severe problem; and level 5, extreme problem [14]. A utility value ranging from 0 to 1 was calculated and defined as the primary outcome of this study. In the Japanese version of the utility value conversion table, “0” indicates death and “1” indicates full health [15]. A hybrid model was prepared by mapping discrete choice experiment (DCE) data onto composite time trade-off data [13].

### Effect of pharmaceutical care for AEs

Moderate or severe (grade ≥ 2) AEs occurring in outpatients receiving chemotherapy triggered a response by physicians and pharmacists to implement a pharmaceutical care intervention based on the clinical practice guidelines [16, 17] or previous reports [18, 19]. At the next visit, the impact of this intervention on the AE was assessed. We investigated the changes in QOL in patients with AEs. QOL was examined at two points, one was the point of the development of the AE [before pharmaceutical intervention] and the other was a point after pharmaceutical intervention for AEs [after pharmaceutical intervention].

### Statistical analysis

Patient demographics were summarized using medians with 25th and 75th percentiles for continuous variables. Frequencies and percentages are shown for categorical variables. AE incidences were also summarized using frequencies and percentages by grade.

The AEs, EQ-5D-5L utility values and all variables used in the analysis were repeated measures from patients.

As primary analysis, we investigated the effect of incidence of AEs (grade ≥ 2) and patient factors on EQ-5D-5L utility values using a mixed effects model. The AEs included in the primary analysis were those with an incidence rate of ≥30%. Distant metastasis [20], neutrophil-lymphocyte ratio (NLR) [21], modified Glasgow prognostic score (mGPS) [21], and CEA [22] are reported to be associated with prognosis in APC. Assuming that these factors are also related to EQ-5D-5L utility value, we performed analysis using the mixed effects model adjusted for distant metastasis, NLR, mGPS, CEA, female gender, age, and time since the start of chemotherapy as covariates. The EQ-5D-5L utility values had a heavily skewed distribution regardless of any conversion. However, we confirmed that there were no problems with the application of the mixed effect model; that is, there was normality in the residuals obtained from the mixed effects model.

For the assessment of the effects of pharmaceutical intervention on AEs with significant association with decreased QOL, we compared the mean EQ-5D-5L utility value using the Wilcoxon signed-rank test before and after pharmaceutical intervention. Regarding adverse events included in the primary analysis, these included adverse events with odds ratios of less than 1 and pharmacological intervention.

Findings with two-sided *p*-values < 0.05 were considered statistically significant. Data were analyzed using the IBM SPSS version 22.0 (IBM Japan Ltd., Tokyo, Japan) and R version 3.6.2 (www.r-project.org).

## Results

### Patients

As shown in Table 1, 59 patients with APC (male: 33, female: 26) received 475 chemotherapy cycles between September 2017 and December 2018 in our outpatient chemotherapy clinic. First-line, 2nd line, 3rd line, preoperative adjuvant and postoperative adjuvant treatments represented 79.6% (378/475), 14.3% (68/475), 2.7% (13/475), 2.9% (14/475) and 0.4% (2/475) of all regimens, respectively. The median time from the start of treatment was 220 days. Distant metastasis was observed in 74.9% (356/475) cases and recurrence was observed in 32.6% (155/475) cases. The most common type of regimen was mFOLFIRINOX (37.7%), followed by GnP (47.8%), and GEM alone (14.5%).

**Table 1:** Patient demographics

	Patients (*n* = 59)		Courses (*n* = 475)	
Gender (male/female)	33/26		307/168	
Age, median (range)	69	(38–84)	69	(38–84)
Albumin (g/dL)	4	(3.7–4.2)	4	(3.7–4.2)
Aspartate aminotransferase (U/L)	24	(19–30)	25	(19–33)
Alanine aminotransferase (U/L)	21	(15–28.5)	20	(14–29)
Serum creatinine (mg/dL)	0.61	(0.51–0.76)	0.63	(0.52–0.80)
Total bilirubin (mg/dL)	0.5	(0.4–0.7)	0.5	(0.4–0.6)
C-reactive protein (CRP, mg/dL)	0.3	(0.06–1.13)	0.22	(0.08–0.65)
Neutrophils (/μL)	2570	(1735–3298)	2510	(1760–3480)
White blood cells (/μL)	4250	(3155–5255)	4400	(3485–5905)
Hemoglobin (g/dL)	10.6	(9.9–11.5)	10.6	(9.7–116)
Platelets (/μL)	17.5	(12.6–26.1)	18.95	(12.9–29.3)
Neutrophil/lymphocyte ratio (NLR)	1.91	(1.41–3.18)	1.99	(1.39–3.12)
Modified Glasgow prognostic score (mGPS, 0/1/2)	43/9/7		376/58/21	
Carcinoembryonic antigen (CEA, ng/mL)	6.2	(2.7–9.7)	4.6	(2.8–8.3)
Carbohydrate antigen 19–9 (CA19–9, U/mL)	655.1	(54.0–2691)	166.1	(39.8–1228)
Chemotherapy, N (%)				
FOLFIRINOX	20	(33.9%)	179	(37.7%)
Gemcitabine plus nab-paclitaxel (GnP)	27	(45.8%)	227	(47.8%)
Gemcitabine monotherapy	12	(20.3%)	69	(14.5%)
Chemotherapy setting, N (%)				
1st line chemotherapy	44	(74.6%)	378	(79.6%)
2nd line chemotherapy	10	(16.9%)	68	(14.3%)
3rd line chemotherapy	2	(3.4%)	13	(2.7%)
Preoperative adjuvant chemotherapy	1	(1.7%)	14	(2.9%)
Postoperative adjuvant chemotherapy	2	(3.4%)	2	(0.4%)
Time from start of first-line chemotherapy (days)	71	(35.5–253.5)	220	(112–386)
Distant metastasis, N (%)	43	(72.9%)	356	(74.9%)
Recurrent cancer, N (%)	15	(25.4%)	155	(32.6%)

All data indicate median, inter-quartile range (25–75th percentiles) unless otherwise indicated

### Proportional incidences of AEs

The proportional incidences of AEs are shown in Table 2. The most common AE of all grades was peripheral neuropathy (73.7%), followed by fatigue (61.3%), anorexia (50.9%), dysgeusia (49.9%), pain (32.8%), and nausea (32.0%). The most common moderate or severe (Grade ≥ 2) AE was also peripheral neuropathy (32.0%), followed by dysgeusia (20.4%), anorexia (17.3%), fatigue (14.3%), pain (14.1%), and alopecia (11.8%).

**Table 2:** Incidence of AEs

	Grade 1	Grade 2	Grade 3	Grade ≥ 2
Peripheral neuropathy	41.7%	31.2%	0.8%	32.0%
Fatigue	46.9%	14.3%	0.0%	14.3%
Anorexia	33.7%	17.3%	0.0%	17.3%
Dysgeusia	29.5%	20.4%	0.0%	20.4%
Pain	18.7%	14.1%	0.0%	14.1%
Nausea	25.1%	6.7%	0.2%	6.9%
Diarrhea	23.4%	1.5%	0.2%	1.7%
Oral mucositis	20.8%	1.3%	0.0%	1.3%
Arthralgia	14.5%	2.5%	0.0%	2.5%
Alopecia	2.9%	11.8%	0.0%	11.8%
Myalgia	5.3%	2.7%	0.0%	2.7%

All courses of chemotherapy were tabulated as a total number

### Risk analysis of QOL deterioration

The median EQ-5D-5L utility value of all enrolled patients was 0.820. The mean EQ-5D-5L utility value for each patient visit is shown in Table 3. The association between QOL and AEs is shown in Table 4. The analysis revealed AEs such as anorexia (Coefficient = − 0.043, 95% CI = − 0.067 – -0.019, *P* = 0.001], pain (Coefficient = − 0.051, 95% CI = − 0.083 – -0.019, *P* = 0.002) and peripheral neuropathy (Coefficient = − 0.021, 95% CI = − 0.041 – -0.002, *P* = 0.035) lowered QOL significantly and independently. Nausea tended to have lower QOL utility values.

**Table 3:** The EQ5D utility value and 5 dimensions of the EuroQol 5 Dimension 5 Level questionnaire of patients

Utility value, median (25–75th percentiles)	0.820 (0.687–0.891)				
5 dimensions	1	2	3	4	5
Mobility, N (%)	239 (50.3%)	192 (40.4%)	34 (7.2%)	8 (1.7%)	0 (0%)
Personal care, N (%)	366 (77.1%)	82 (17.3%)	20 (4.2%)	5 (1.1%)	0 (0%)
Usual activities, N (%)	259 (54.5%)	161 (33.9%)	40 (8.4%)	9 (1.9%)	2 (0.4%)
Pain/discomfort, N (%)	177 (37.3%)	248 (52.2%)	36 (7.6%)	11 (2.3%)	0 (0%)
Anxiety/depression, N (%)	250 (52.6%)	180 (37.9%)	26 (5.5%)	15 (3.2%)	0 (0%)

**Table 4:** Analysis using a mixed effects model for AEs and risk factors associated with the EQ-5D-5L utility value in patients who received chemotherapy

Factors	Coefficient	95% CI	*P* value
Nausea	−0.015	−0.046 – 0.015	0.339
Anorexia	−0.043	−0.067 – − 0.019	0.001
Pain	−0.051	− 0.083 – − 0.019	0.002
Fatigue	− 0.001	− 0.025 – 0.024	0.966
Peripheral neuropathy	−0.021	− 0.041 – 0.002	0.035
Dysgeusia	0.015	−0.007 – 0.038	0.197
Female	−0.022	−0.092 – 0.047	0.543
Age (IQR:61.5–75)	−0.016	−0.065 – 0.032	0.524
mGPS (IQR:0–2)	−0.003	−0.034 – 0.028	0.870
Distant metastasis	−0.013	−0.067 – 0.039	0.626
FOLFIRINOX	0.021	−0.026 – 0.068	0.400
NLR (IQR:1.4–3.1)	0.002	−0.005 – 0.009	0.603
CEA, μg/L (IQR:2.8–8.3)	−0.001	−0.002 – 0	0.125
Time from start of first-line chemotherapy, day (IQR:112–386)	−0.007	−0.023 – 0.008	0.365

*mGPS* modified Glasgow prognostic score, *NLR* Neutrophil-lymphocyte ratio, *CEA* Carcinoembryonic antigen, *IQR* Inter-quartile range (25–75th percentiles)

Coefficient for continuous variables indicate the difference of mean in QOL for the increment in IQR

### Changes in EQ-5D-5L utility values after pharmaceutical intervention

**Fig. 1:**
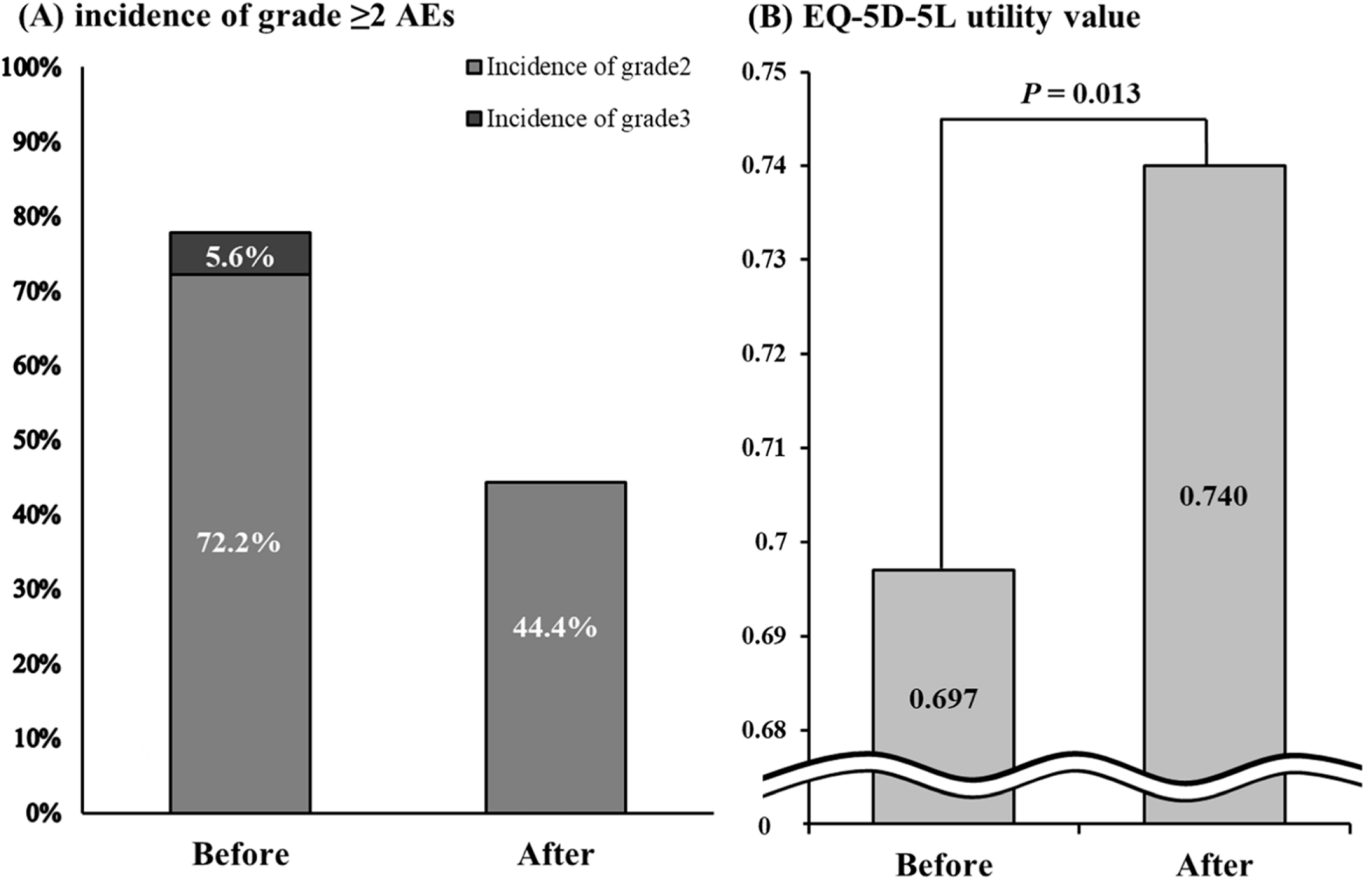
Comparison of the incidence of grade ≥ 2 AEs affecting QOL (i.e., nausea, peripheral neuropathy, pain) (**A**) and the average of EQ-5D-5 L utility value (**B**), before and after pharmaceutical intervention (B: Wilcoxon signed-rank test)

Of patients experiencing AEs including nausea, peripheral neuropathy, and pain, 18 patients underwent pharmaceutical intervention. We evaluated the degree of change in the EQ-5D-5L utility value between pre-intervention and post-intervention. The details of pharmaceutical interventions are shown in Table 5. For nausea, oral administration of prochlorperazine (22.2%), olanzapine (22.2%) and granisetron (5.6%) were added. The oral administration of pregabalin (16.7%) and duloxetine (16.7%) was used to treat peripheral neuropathy, and the oral administration of acetaminophen (5.6%), loxoprofen (5.6%), and tapentadol (5.6%) was used for pain. As shown in Fig. 1, the EQ-5D-5L utility value significantly improved.

**Table 5:** Pharmaceutical interventions for grade ≥ 2 AEs with highest incidence

Adverse events (N)	Interventions (N)
Nausea (9)	Prochlorperazine (4), olanzapine (4), granisetron (1)
Peripheral neuropathy (6)	Pregabalin (3), duloxetine (3)
Pain (3)	Acetaminophen (1), loxoprofen (1), tapentadol (1)

All interventions were tabulated for each course of chemotherapy

## Discussion

We conducted a retrospective analysis to investigate the impact of current outpatient chemotherapy-related AEs on QOL in patients with APC. Primary analysis revealed that anorexia, pain and peripheral neuropathy were the independent and significant AEs for reducing QOL. Nausea tended to have lower QOL utility values.

Tachi et al. showed that, in breast cancer patients receiving outpatient chemotherapy, patients with anorexia had significantly lower EQ-5D utility values and total QOL-ACD scores than those without anorexia [9]. Anorexia and nausea worsen nutritional status and lead to a decrease in muscle mass. Onishi et al. revealed that there were significant associations between sarcopenia and QOL decline in patients with digestive diseases [23]. In APC patients, malnutrition and sarcopenia are well-known factors associated with limited chemotherapy tolerance and poor QOL [24, 25]. Pancreatic enzyme replacement therapy [24] and nutritional therapy are important for patients with APC early in the course of chemotherapy.

Consistent with our present findings of a relationship between pain and QOL, a previous study demonstrated that the Functional Assessment of Cancer Therapy-General (FACT-G) score in elderly patients who received chemotherapy and radiation therapy was significantly decreased by pain, particularly with regard to functional score [26]. In general, patients with APC develop pain in the early stage of disease onset due to tumor invasion and neurodegeneration. As almost all patients develop severe pain by the end of life, it is assumed that many patients in this study developed pain and experienced a deterioration in QOL.

Hershman et al. evaluated the relationship between peripheral neuropathy and QOL in breast cancer patients who received paclitaxel treatment and reported that peripheral neuropathy was a significant factor in the deterioration of QOL, especially in physical and functional aspects [27]. Lu et al. investigated the association among peripheral neuropathy, restriction of daily activity, and QOL in colorectal cancer patients who developed peripheral neuropathy caused by oxaliplatin, and reported peripheral neuropathy to be a confounder of the relationships among restriction of daily activity, mood, and QOL [28]. In their systematic review, Girach et al. reported that painful CIPN independently affects overall patient QoL although general health was rated as high [29]. Oxaliplatin in the FOLFIRINOX regimen and Nab-paclitaxel in the GnP regimen had a higher incidence of peripheral neuropathy, and it was considered that these peripheral neuropathies were associated with the lower QOL of patients with APC in this study.

The present results are consistent with those of Akhtari-Zavare M et al. who also analyzed the association between the presence of nausea and QOL in cancer patients using the WHOQOL-BREF, and showed that nausea negatively influenced QOL in each component of physical, psychological, environmental and social relationships [30]. The present study also suggested that nausea caused by FOLFORINOX, a highly emetogenic risk regimen, and GnP therapy, a moderately emetogenic risk regimen, may affect QOL. In our outpatient cancer chemotherapy clinic, pharmacists provide input in collaboration with doctors for chemotherapy-induced AEs such as nausea, pain, and peripheral neuropathy. The EQ-5D-5L utility value was significantly improved after pharmaceutical intervention (pre-intervention: 0.697, post-intervention: 0.740, *P* = 0.013). Nausea, pain, and peripheral neuropathy can be expected to improve QOL through intervention based on guidelines [16, 17] or previous reports [18, 19].

There are four limitations in the present study. First, this study was a retrospective study with a small sample size. Therefore, confounding may not have been completely excluded. Second, as we could not compare with a control group that did not receive chemotherapy for obvious ethical reasons, it is not completely clear whether the deterioration in QOL was due to chemotherapy and the AEs or the disease. Third, since EQ-5D-5L does not have the objective Minimally Important Difference [31], it may not explain the clinical significance in QOL by the differences in QOL score. Fourth, it is difficult to distinguish strictly between events caused by patient’s clinical condition and those caused by chemotherapy.

## Conclusion

We found that AEs such as anorexia, pain and peripheral neuropathy were independent and significant factors for reduction of QOL in patients with APC receiving outpatient chemotherapy. From this result, when performing pharmaceutical interventions in APC chemotherapy, it is assumed that appropriate pharmaceutical intervention with particular attention to these AEs can effectively improve QOL of patients with APC receiving outpatient chemotherapy.

## Data Availability

Not applicable.
